# Colonization of Toxigenic *Clostridium difficile* Among Intensive Care Unit Patients: A Multi-Centre Cross-Sectional Study

**DOI:** 10.3389/fcimb.2020.00012

**Published:** 2020-01-30

**Authors:** Hongfei Mi, Rong Bao, Yao Xiao, Yangwen Cui, Wei Sun, Yan Shen, Qingfeng Shi, Xiang Chen, Jiabing Lin, Bijie Hu, Xiaodong Gao

**Affiliations:** ^1^Xiamen Branch, Zhongshan Hospital, Fudan University, Xiamen, China; ^2^Zhongshan Hospital, Fudan University, Shanghai, China; ^3^Zhongshan Hospital, School of Medicine, Xiamen University, Xiamen, China; ^4^Xiamen Hospital of Traditional Chinese Medicine, Xiamen, China

**Keywords:** *Clostridium difficile*, colonization, toxigenic, infection control, cross-sectional study

## Abstract

**Background:** *Clostridium difficile* (CD) is a major cause of healthcare-associated infections and antibiotic-associated diarrhea in hospitalized patients worldwide. Carriers of toxigenic CD (tCD) have a higher risk of developing CD infections and can transmit CD to the environment and susceptible patients. However, little is known regarding the carriers and transmission of tCD in China.

**Methods:** A multi-center cross-sectional study of tCD colonization (tCDC) was conducted from October 24 to 31, 2014, at 33 hospitals in Shanghai, China. Rectal swabs or stool samples were collected and tested, and the clinical and demographic status, epidemiological data, and blood parameters of 531 participants were recorded. The status of tCDC was defined by a positive result on the nucleic acid amplification test for the *tcdA* (toxin A), *tcdB* (toxin B), and *cdtAB* (toxin CDT) genes after positive bacterial culture.

**Results:** The overall prevalence of CD colonization (CDC) was 19.02%, tCDC accounted for 92.08%, and A+B+CDT– was the dominant genotype (87.13%). The CD infection (CDI) prevalence was 1.51%. Potential tCDC-associated factors were admission to secondary grade hospitals, a body mass index <18.5, hospitalization during the previous 30 days, underlying diseases (including hypertension, diabetes mellitus, coronary heart disease, and respiratory failure), diarrhea during the previous 7 days, and exposure to fluoroquinolones or lansoprazole.

**Conclusions:** This study reveals the prevalence of CDC and tCDC in Shanghai, elucidates several associated factors, contributes to the awareness of the current epidemiology in parts of eastern China and provides new insights for further study and infection control practices.

## Introduction

*Clostridium difficile* infection (CDI) is a persistent clinical challenge for the past four decades, as it has been a worldwide healthcare-associated infection and the major cause of antibiotic-associated diarrhea in hospitalized patients (McFarland et al., 2018). The clinical features vary from no symptoms (asymptomatic), infectious diarrhea, pseudomembranous colitis, toxic megacolon to even death (Ghose, 2013; Steele et al., 2015; Shoaei et al., 2019). Global attention has recently been directed toward the incidence of CD infection (CDI); despite efforts to prevent patient deterioration, CDI results in a worrisome outcome, and it is associated with increased morbidity, mortality, medical costs, and family burdens (Lin et al., 2015; Deshpande et al., 2017; Ho et al., 2017; Peng et al., 2018). In the United States, CDI prolongs hospital stays by 2.8–5.5 days, increases medical costs by $3,006–$15,397 per episode, and results in mortality in 5–10% of cases (Dubberke et al., 2016).

Risk factors for susceptible patients include the following: (a) age over 65 years old, (b) a long duration of hospitalization, (c) a history of prior hospitalization, (d) antimicrobial exposure (especially broad-spectrum second- or third-generation cephalosporins, penicillins, clindamycin, and fluoroquinolones), (e) a history of taking proton pump inhibitors (PPIs) or other antacid treatments, (f) severe illness, and (g) immune suppression (Vonberg et al., 2008; Janarthanan et al., 2012; Nissle et al., 2016). It should be noted that patients carrying toxigenic CD (tCD) upon hospital admission have a risk of subsequent CDI almost 6 times higher than that of non-carriers (Zacharioudakis et al., 2015), and carriers of tCD may become significant reservoirs for transmission to the environment and susceptible patients mainly via direct or indirect contact (Curry et al., 2013; Ghose, 2013). Therefore, to prevent nosocomial CDI transmission, the early recognition of tCD colonization (tCDC) upon admission is essential for the timely implementation of infection control measures, antibiotic stewardship measures, contact isolation precautions, proper hand hygiene procedures, environmental cleaning and disinfection procedures, etc. (Yakob et al., 2014; McDonald et al., 2018). A meta-analysis concluded that the overall pooled CD positivity rate among diarrhea patients was 14.8%, with a higher prevalence in East Asia (19.5%) than in South Asia (10.5%) or the Middle East (11.1%) (Deshpande et al., 2017). Routine detection methods of CD includes toxigenic culture, cell cytotoxic neutralization assay, glutamate dehydrogenase assay, enzyme immunoassays, nucleic acid amplification tests, etc. (Martinez-Melendez et al., 2017). However, the popularization of these methods may be problematic in developing countries, likely due to limitations in awareness, laboratory capacity and capabilities, and surveillance systems (Collins et al., 2013; Forrester et al., 2017). As a result, information on carriers and transmission of tCD in China is scarce. Hence, we conducted a multi-center cross-sectional study to reveal the prevalence of tCDC among intensive care unit (ICU)-hospitalized patients.

## Materials and Methods

### Study Population and Data Collection

A multi-center cross-sectional study was conducted in ICUs from 33 public general hospitals (including 15 tertiary grade A units, 4 tertiary grade B units, and 14 secondary grade units) in Shanghai. Surveys in different hospitals were organized respectively on a single day between Oct 24 and 31, 2014. Hospitals were classified according to National Hospital Grade Accreditation that tertiary hospitals were equipped over 500 beds while secondary were inferior. Subjects were eligible for inclusion if they were over 14 years old and were in one of the study ICUs during the study period. The exclusion criteria were as follows: (a) the patient had incomplete medical records; or (b) redundant data from patients who received repeated inquiry or specimen collection. Patients who may have been on treatment at the time of surveillance for CDI were not excluded, since the CD test may remain positive for a long as 30 days in patients who have resolution of symptoms (Surawicz et al., 2013). A total of 555 patients were hospitalized in the study sites on the survey days, of which 24 patients under 14 years old were excluded. Underlying diseases of the study participants were diagnosed by clinicians in charge, according to the International Classification of Diseases 10th Revision. Stool specimen or rectal swab from single patient was collected on the survey day (rectal swab was sampled from patient with ileus, which had been proven to be equivalent with stool for laboratory test) (Kundrapu et al., 2012). Clinical and demographic status, epidemiological data, and blood parameters (white blood cell counts and creatinine levels) were recorded simultaneously by trained infection control agents, and rechecked by YC and WS, antimicrobial application for perioperative prophylaxis was not collected.

### Diagnostic Criteria

The diagnosis of CDI was based on a combination of the following clinical criteria (Gerding and Johoson, 2017): (1) diarrhea (≥3 unformed stools per 24 h for over 2 days) with no other recognized cause; and (2) toxin A or B detected in the stool, toxin-producing *C. difficile* detected in the stool by PCR or culture, or pseudomembranes seen in the colon. In this study, the history of diarrhea was defined as the occurrence of symptoms that ≥3 unformed stools per 24 h for over 2 days according to the *Stool Form Scale* (Lewis and Heaton, 1997) within 7 days prior to the survey.

### Laboratory Tests

CDI symptoms are mostly mediated by toxins, toxin A (TcdA, enterotoxin, encoded by *tcdA*), toxin B (TcdB, cytotoxin, encoded by *tcdB*), and binary toxin (CDT, encoded by *cdtAB*), have been identified as the major virulence factors (Dayananda and Wilcox, 2019; Shoaei et al., 2019).

All stool and rectal swab samples collected from 33 hospitals were transported by anaerobe transport culture medium (Hopebio, China) at room temperature to the central laboratory in Zhongshan Hospital, Fudan University for subsequent tests. The specimens undergoing CD culture were treated with 75% (v/v) ethanol at a 1:1 ratio for 1 h before inoculation onto ChromID *C. difficile* agar (CDIF, BioMerieux SA, France), whose sensitivity and specificity were higher than BBL *C. difficile* selective agar (Han et al., 2014). All agar plates were incubated anaerobically at 37°C for 72 h before identification and DNA extraction. CD were identified by matrix-assisted laser desorption/ionization time-of-flight mass spectrometry (BioMerieux SA, France). Positive CD cultures were analyzed by PCR for gene *tcdA, tcdB*, and *cdtAB*. DNA from all CD-positive cultures was extracted and purified using the QIAcube automatic nucleic acid purifier and QIAamp DNA blood kit (Qiagen, Germany) and stored at −80°C until testing. PCR primers sequences were published by Jin et al. (2016). CD ATCC 43255 (A+B+CDT–, RT 087) was used as a control strain.

### Statistical Analyses

The proportions of laboratory-confirmed tCDC among patients with different clinical and demographic characteristics were calculated. The Mann–Whitney *U*-test was used for continuous variables with skewed distributions, and a χ^2^ test or Fisher's exact test was used for categorical variables. The odds ratios (ORs) with 95% confidence intervals (CIs) were calculated among compared groups to assess the odds of developing tCDC. The statistical analyses were performed using SPSS 19.0 for Windows (SPSS Inc., Chicago, Illinois, USA). A two-sided *P*-value of <0.05 was considered statistically significant.

## Results

### Demographic Characteristics of the Study Subjects

Among the 531 study participants, the CDC prevalence was 19.02% (101/531), and the tCDC accounted for 92.08% (93/101). The *tcdA, tcdB*, and *cdtAB* genes were tested in all CDC subjects, and all subjects were *cdtAB* negative. The CDC genotypes were A–B–CDT– (7.92%, 8/101), A+B+CDT– (87.13%, 88/101), and A+B–CDT– (4.95%, 5/101). According to the diagnostic criteria, 8 subjects were diagnosed with CDI, the overall prevalence of CDI was 1.51% (8/531).

The subjects were recruited from tertiary grade A hospitals (274/531), tertiary grade B hospitals (100/531) and secondary grade hospitals (157/531). Patients hospitalized in secondary grade hospitals were more likely to acquire tCDC than those in tertiary grade A hospitals (OR = 1.848; 95% CI, 1.114–3.064; *P* = 0.017). The sex ratio (male: female) of the subjects was approximately 1.3:1, and the prevalence of tCDC was 18.15% (55/303) in males and 16.67% (38/228) in females (*P* = 0.656). The median age of the participants was 74 years old. Compared with the relatively younger group, patients aged over 65 years were more likely to present tCDC (OR = 1.792; 95% CI, 1.041–3.083; *P* = 0.034). The body mass indexes (BMIs) were investigated for 235 study subjects; the BMI data were unavailable for the remaining 296 patients due to critical conditions (these patients remained bedridden during their hospital stay). Patients with a BMI < 18.5 were more likely to acquire tCDC than patients with a normal BMI (OR = 2.605; 95% CI, 1.114–6.091; *P* = 0.027) (Table 1).

**Table 1:** Demographic characteristics of the study subjects.

**Variables**	***N***	***n***	**Prevalence (%)**	***P*^**a**^**	**OR (95% CI)**	***P*^**b**^**
**Hospital classification**				0.053		
Tertiary grade A	274	38	13.9		Ref.	
Tertiary grade B	100	19	19.0		1.457 (0.795–2.670)	0.223
Secondary grade	157	36	22.9		1.848 (1.114–3.064)	**0.017**
**Gender**				0.656		
Female	228	38	16.7		Ref.	
Male	303	55	18.2		1.109 (0.704–1.747)	0.656
**Age (years)**				**0.034**		
15–64	157	19	12.1		Ref.	
≥65	374	74	19.8		1.792 (1.041–3.083)	**0.034**
**Body mass index**				0.169		
18.5–23.9	123	24	19.5		Ref.	
<18.5	31	12	38.7		2.605 (1.114–6.091)	**0.027**
24–28	62	15	24.2		1.316 (0.633–2.739)	0.462
>28	19	4	21.0		1.100 (0.335–3.615)	0.875

*OR, odds ratio; CI, confidence interval; Ref., reference*.

*P^a^, Comparison among all the subgroups; P^b^, Comparison with the reference subgroup; numbers in bold indicate statistical significance*.

### Clinical Conditions of the Study Subjects

Clinical conditions were investigated for all the study subjects (Table 2). In patients hospitalized during the previous 30 days, the prevalence of tCDC was higher than that in those who were not (23.36 vs. 15.48%, *P* = 0.037). The prevalence of tCDC was increased in patients with hypertension (23.33 vs. 12.71%, *P* = 0.001), diabetes mellitus (25.00 vs. 15.60%, *P* = 0.022), coronary heart disease (28.83 vs. 14.52%, *P* < 0.001), and respiratory failure (28.57 vs. 16.56%, *P* = 0.049). The prevalence of tCDC in patients who experienced diarrhea during the previous 7 days was higher than those who did not (35.71 vs. 16.50%, *P* = 0.018). There were no significant differences in the prevalence of tCDC between groups considering pulmonary infection, renal failure, gastric tube insertion, fever, history of stroke, chronic digestive disorders, laparotomy, thoracotomy, craniotomy, or orthopedic characteristics (*P* > 0.05). No significant difference was shown in peripheral white blood cell counts and creatinine levels between tCDC patients and others (data not shown).

**Table 2:** Clinical conditions of the study subjects.

**Variables**	***N***	***n***	**Prevalence (%)**	**OR (95% CI)**	***P***
**Hospitalization during the previous 30 days**					**0.037**
No	394	61	15.5	Ref.	
Yes	137	32	23.4	1.664 (1.029–2.691)	
**Hypertension**					**0.001**
No	291	37	12.7	Ref.	
Yes	240	56	23.3	2.089 (1.324–3.298)	
**Diabetes Mellitus**					**0.022**
No	423	66	15.6	Ref.	
Yes	108	27	25.0	1.803 (1.084–2.998)	
**Pulmonary infection**					0.411
No	422	71	16.8	Ref.	
Yes	109	22	20.2	1.250 (0.734–2.130)	
**Coronary heart disease**					**<0.001**
No	420	61	14.5	Ref.	
Yes	111	32	28.8	2.384 (1.457–3.900)	
**Respiratory failure**					**0.049**
No	489	81	16.6	Ref.	
Yes	42	12	28.6	2.015 (0.990–4.101)	
**Renal failure**					0.106
No	513	87	17.0	Ref.	
Yes	18	6	33.3	2.448 (0.895–6.700)	
**History of stroke**					0.301
No	426	71	16.7	Ref.	
Yes	105	22	21.0	1.325 (0.776–2.262)	
**History of chronic digestive disorders**					0.305
No	503	86	17.1	Ref.	
Yes	28	7	25.0	1.616 (0.666–3.921)	
**Gastric tube insertion during the previous 30 days**					0.522
No	307	51	16.6	Ref.	
Yes	224	42	18.8	1.158 (0.738–1.817)	
**Diarrhea during the previous 7 days**					**0.018**
No	503	83	16.5	Ref.	
Yes	28	10	35.7	2.811 (1.253–6.307)	
**Fever (body temperature ≥ 38.0°C) during the previous 7 days**					0.809
No	354	61	17.2	Ref.	
Yes	177	32	18.1	1.060 (0.661–1.699)	
**History of laparotomy**					0.689
No	496	86	17.3	Ref.	
Yes	35	7	20.0	1.192 (0.504–2.817)	
**History of thoracotomy**					0.086
No	516	93	18.0		
Yes	15	0	0.0		
**History of craniotomy**					0.516
No	514	89	17.3	Ref.	
Yes	17	4	23.5	1.469 (0.468–4.611)	
**History of orthopedic**					0.749
No	514	91	17.7	Ref.	
Yes	17	2	11.8	0.620 (0.139–2.757)	

*OR, odds ratio; CI, confidence interval; Ref., reference*.

*Numbers in bold indicate statistical significance*.

### Drug Exposure of the Study Subjects

Antibiotic and PPI exposure over the previous 30 days were investigated for all subjects (Table 3). The prevalence of tCDC in patients who were treated with over 4 kinds of antibiotics tended to be higher than those who received no antibiotics treatment (25.93 vs. 14.81%, OR = 2.013, 95% CI, 0.930–4.355, *P* = 0.076). The prevalence of tCDC was increased in patients who underwent treatment with fluoroquinolones (23.89 vs. 15.79%, OR = 1.674, 95% CI, 1.009–2.777, *P* = 0.044) and lansoprazole (28.26 vs. 16.49%, OR = 1.994, 95% CI, 1.005–3.957, *P* = 0.045). There were no significant differences in the prevalence of tCDC considering treatment with other antibiotic treatment.

**Table 3:** Drug exposure of the study subjects.

**Variables**	***N***	***n***	**Prevalence (%)**	**OR (95% CI)**	***P***
**Antibiotics exposure during the previous 30 days**					0.339
No	135	20	14.8	Ref.	
Yes	396	73	18.4	1.300 (0.758–2.227)	
Kinds of Antibiotics					0.072
0	135	20	14.8	Ref.	
1	168	37	22.0	1.624 (0.892–2.956)	0.112
2	115	15	13.0	0.863 (0.419–1.774)	0.688
3	59	7	11.9	0.774 (0.308–1.944)	0.586
≥4	54	14	25.9	2.013 (0.930–4.355)	0.076
**β-lactamase inhibitors**					
Total					0.951
No	427	75	17.6	Ref.	
Yes	104	18	17.3	0.982 (0.558–1.730)	
**Cefoperazone Sodium and Sulbactam Sodium**					0.158
No	482	88	18.2	Ref.	
Yes	49	5	10.2	0.509 (0.196–1.320)	
Piperacillin and tazobactam					0.467
No	479	82	17.1	Ref.	
Yes	52	11	21.2	1.299 (0.641–2.633)	
**Carbapenems**					
Total					0.335
No	430	72	16.7	Ref.	
Yes	101	21	20.8	1.305 (0.758–2.247)	
Meropenem					0.785
No	489	85	17.4	Ref.	
Yes	42	8	19.1	1.118 (0.500–2.501)	
Imipenem					0.207
No	476	80	16.8	Ref.	
Yes	55	13	23.6	1.532 (0.786–2.985)	
**Fluroquinolones**					
Total					**0.044**
No	418	66	15.8	Ref.	
Yes	113	27	23.9	1.674 (1.009–2.777)	
Levofloxacin					**0.026**
No	474	77	16.2	Ref.	
Yes	57	16	28.1	2.012 (1.075–3.767)	
Moxifloxacin					1.000
No	504	89	17.7	Ref.	
Yes	27	4	14.8	0.811 (0.274–2.403)	
**Pyrroles**					0.787
No	506	88	17.4	Ref.	
Yes	25	5	20.0	1.188 (0.434–3.249)	
**Nitroimidazoles**					
Total					0.200
No	503	91	18.1	Ref.	
Yes	28	2	7.1	0.348 (0.081–1.494)	
Ornidazole					0.702
No	519	92	17.7	Ref.	
Yes	12	1	8.3	0.422 (0.054–3.309)	
Metronidazole					0.338
No	513	92	17.9	Ref.	
Yes	18	1	5.6	0.269 (0.035–2.048)	
**Cephalosproins**					
Total					0.393
No	345	64	18.6	Ref.	
Yes	186	29	15.6	0.811 (0.502–1.311)	
C4G					0.813
No	483	84	17.4	Ref.	
Yes	48	9	18.8	1.096 (0.512–2.349)	
C3G					0.958
No	452	79	17.5	Ref.	
Yes	79	14	17.7	1.017 (0.544–1.903)	
C2G					0.093
No	463	86	18.6	Ref.	
Yes	68	7	10.3	0.503 (0.222–1.138)	
C1G					0.538
No	527	92	17.5	Ref.	
Yes	4	1	25.0	1.576 (0.162–15.322)	
**Proton pump inhibitors exposure during the previous 30 days**					
Total					0.864
No	358	62	17.3	Ref.	
Yes	173	31	17.9	1.042 (0.648–1.676)	
Omeprazole					0.165
No	462	85	18.4	Ref.	
Yes	69	8	11.6	0.582 (0.268–1.261)	
Pantoprazole					0.818
No	482	85	17.6	Ref.	
Yes	49	8	16.3	0.911 (0.412–2.014)	
Lansoprazole					**0.045**
No	485	80	16.5	Ref.	
Yes	46	13	28.3	1.994 (1.005–3.957)	

*OR, odds ratio; CI, confidence interval; Ref., reference; C4G, fourth generation cephalosporin; C3G, third generation cephalosporin; C2G, second generation cephalosporin; C1G, first generation cephalosporin. Numbers in bold indicate statistical significance*.

## Discussion

A better understanding of the epidemiological characteristics of tCDC will contribute to the control and prevention of healthcare-associated infections and antibiotic-associated diarrhea. The present study investigated the prevalence of tCDC under different medical conditions among ICU patients in multiple medical institutions. The results showed a prevalence of 19.02% for CDC, 17.51% for tCDC, and 1.51% for CDI overall. Two studies conducted among ICU patients in China showed that the tCDC rates were 17.81 and 16.67%, and the CDI rates were 2.90 and 2.99%, respectively (Hung et al., 2012; Lin et al., 2015). While rates of tCDC of overall inpatients were about 10% (Behar et al., 2017; Jin et al., 2017). ICU patients may be at higher risk of tCDC on account of long-term hospitalization and severe illness. Patients in secondary grade hospitals, patients with a BMI < 18.5, patients who were hospitalized during the previous 30 days, patients who had underlying diseases (hypertension, diabetes mellitus, coronary heart disease, and respiratory failure), patients who experienced diarrhea during the previous 7 days, and patients who were administered fluoroquinolones or lansoprazole had an increased prevalence of tCDC.

In this cross-sectional study, the major genotype for CDC was A+B+CDT– (87.13%), but no A–B+CDT– isolates, which were previously reported to be epidemic in Asia including parts of China, were detected (Collins et al., 2013; Cheng et al., 2016; Jin et al., 2017), showing significant regional variation in the genotype distribution of CD. Similarly, no CDT positive strains were detected, neither in studies conducted in northern China (Jin et al., 2017; Wang et al., 2018).

CDI occurs frequently in hospitals with a high level of antimicrobial use; often, the environment is contaminated by CD spores (Ghose, 2013; Gerding and Johoson, 2017). In this study, the prevalence of tCDC was relatively lower in the tertiary grade hospitals than that in the secondary grade hospitals. There were more advanced medical resources and specialists in the tertiary grade hospitals; therefore, tertiary grade hospitals may provide more professional medical care and sustain a better antibiotic stewardship programme, which is strongly recommended to minimize unnecessary high-risk antibiotic administration and the number of antibiotic agents prescribed to reduce CDI risk (McDonald et al., 2018). Moreover, education for cleaning personnel of regular environmental cleaning and disinfection is essential (Vonberg et al., 2008). On the other hand, patients who are admitted to secondary grade hospitals are usually located in the suburbs, suggesting their poor economic capabilities and decreased concerns about health, which may result in a higher susceptibility to infections to some extent.

The prevalence of tCDC was also increased in underweight patients (BMI < 18.5). Underweight could reflect malnutrition and was noted to be an independent predictor of tCDC (Behar et al., 2017), which may increase susceptibility to infections. Moreover, underweight may be a reflection of poor economic conditions, which results in poor hygienic status. The relationship between underweight and risk of infection was most prevalent in developing countries (Dobner and Kaser, 2018).

Our results showed an increased prevalence (23.36%) of tCDC in patients who were hospitalized during the previous 30 days, as well as in patients with underlying diseases (including hypertension, diabetes mellitus, coronary heart disease, and respiratory failure). A previous hospital stay was shown to be a risk factor for CDC (Behar et al., 2017), and it could be inferred that frequent contact with the nosocomial environment increased the odds of infection. Additionally, a previous hospital stay may represent a poor health condition or an underlying disease, increasing the susceptibility to tCDC.

An increased prevalence of tCDC was observed in patients who experienced diarrhea during the previous 7 days. Diarrhea is a clinical symptom of CDI (Schaffler and Breitruck, 2018); the differential diagnosis of diarrhea should include CDI, and CDI-associated potential life-threatening conditions, such as pseudomembranous colitis or toxic megacolon, should receive increased attention. The early detection of CD and CD toxins is critical, as this allows earlier treatment that can significantly reduce the morbidity, mortality, medical cost, and family burden of CDI. For nosocomial infection prevention and control, the early detection of CD in patients who experienced recent diarrhea would be of great value for early case identification in clinical practice, subsequently followed by isolation, cohort nursing, antimicrobial stewardship, surveillance, education for targeted populations, reinforced environmental disinfection and early therapy to limit the spread of the infection (Vonberg et al., 2008; Yakob et al., 2014).

In addition to exposure to tCD and an inadequate host immune response, exposure to antimicrobial agents, which likely resulted in a disruption of the normal gastrointestinal microbiota, is a key event in the development of CDI (Gerding and Johoson, 2017). Fluoroquinolones have been found to be one of the primary precipitating antimicrobials associated with CDI, along with broad-spectrum cephalosporins, ampicillin, and clindamycin (Dubberke et al., 2016). According to our results, the prevalence of tCDC was increased in patients previously exposed to fluoroquinolones (especially levofloxacin). Levofloxacin is one of the most commonly used fluoroquinolones and was reported to increase the odds of CDI by 9.3 times (Wong-McClure et al., 2013). Different from previous studies reporting that exposures of broad-spectrum second-, third-generation cephalosporins, or penicillins were risk factors of CDI (Vonberg et al., 2008; Janarthanan et al., 2012; Nissle et al., 2016), no significant association between broad-spectrum cephalosporins and tCDC was observed in this study. This difference may be interpreted by a cross-sectional study design, of which no causal relationships can be deduced, as well as by a different time interval (30 days) between the antibiotics exposure and the survey day. Additionally, clindamycin is one kind of known high-risk antibiotics, but it was not analyzed since only 4 of 531 subjects received clindamycin treatment.

Several studies demonstrated that PPI use had a clinical association with CDI (Dubberke et al., 2007; Janarthanan et al., 2012; Kwok et al., 2012; Lin et al., 2015), but the mechanism remains unclear since no randomized controlled trials or quasi-experimental studies have studied the relationship between discontinuing or avoiding PPI use and the risk of CDI (McDonald et al., 2018). The relationship between PPI exposure and CDI remains controversy, even a global review of guidelines could not extract a conclusion of it (Balsells et al., 2016). No association between PPI use and tCDC was observed in our study, but interestingly, patients who received lansoprazole treatment acquired tCDC more often than patients who did not (28.26 vs. 16.49%, OR = 1.994, 95% CI, 1.005–3.957). Lansoprazole was previously reported to be associated with tCDC in an experimental study (Kaur et al., 2007). US Food and Drug Administration data also reported 2 signals of disproportionate reporting (SDR) for CD associated with lansoprazole, while no SDRs for omeprazole, pantoprazole and other PPIs were reported (Hauben et al., 2007). Consistent with this monitoring data, our finding provided clues for further study of the relationship between PPI exposure and CDI.

To the best of our knowledge, this is the first multi-center study to report the prevalence of tCDC among ICU patients in Eastern China. However, this study has several limitations that need to be addressed. First, this study was conducted in 2014, the variation trend of CDI or CDC from then on was scarcely possible to estimate due to unachievable popularization of routine CD detection methods in China and a paucity of data reported about CDI or CDC genotype distribution. Additionally, infection control programmes targeting the carbapenem-resistant *A. baumannii* and carbapenem-resistant *Enterobacteriaceae* were continuously implemented in Shanghai in the past decade, and the strategies might have stable influence on the prevalence of CDI or CDC. A longitudinal study would be worthwhile in future to further understand the variation trend of CDI or CDC. Second, tCD was detected using PCR in this study; PCR may be oversensitive because extremely few or insignificant genetic residues were detected. It is hard to distinguish contamination from true colonization. Third, as this study was conducted in ICUs, a considerable part of patients had lost the ability of movement at admission, of whom the height and weight data could not be collected. Thus the association between BMI and tCDC were analyzed in only 44.3% (235/531) of the participants. The demographic characteristics between patients with and without BMI data were compared and showed no significant difference. Thus the missing values of BMI data might have little effect on the conclusion that low BMI was associated with tCDC. Certainly a larger sample size to verify this association is imperative. Fourth, the disease severity of ICU patients was not analyzed due to the lack of a unified evaluation standard in our all study sites. Finally, associations between tCDC and antibiotics were discussed insufficiently. We entered all kinds of documented antibiotics in this study into the factorial analysis, but the sample size for each combination was insufficient to achieve significant results. A larger sample size would be worthy of further study to determine the association between a specific combination of antibiotics and tCDC.

In conclusion, we surveyed the prevalence of CDC, tCDC, and the CDI rate (%) among ICU patients in Shanghai and demonstrated the epidemic genotype distribution of CD. The findings highlighted the prevalence among different hospitals grades, elucidated some risk factors related to tCDC, and provided new insights for further study and infection control practices. To prevent the spread of nosocomial CDI, awareness of the epidemiology, risk factors and infection control strategies should be increased reinforced.

## Data Availability Statement

All datasets generated for this study are included in the article.

## Ethics Statement

This study was approved by the Ethics Committee of Zhongshan Hospital, Fudan University, and was in compliance with national legislation and the Declaration of Helsinki guidelines. Written informed consent was obtained from the patient or, from next of kin or an independent patient advocate if the patient lacked this capacity.

## Author Contributions

HM, RB, and YX wrote the main manuscript text. YC and WS collected samples and information of study subjects. YS and QS contributed to laboratory tests. XC and JL did the statistical analysis. BH and XG conceived the study and revised the manuscript.

### Conflict of Interest

The authors declare that the research was conducted in the absence of any commercial or financial relationships that could be construed as a potential conflict of interest.
